# A Systematic Review of NAFLD-Associated Extrahepatic Disorders in Youths

**DOI:** 10.3390/jcm8060868

**Published:** 2019-06-17

**Authors:** Lucia Pacifico, Francesco Massimo Perla, Mario Roggini, Gianmarco Andreoli, Miriam D’Avanzo, Claudio Chiesa

**Affiliations:** 1 Department of Pediatrics, Sapienza University of Rome, 00161 Rome, Italy; francescomassimo.perla@uniroma1.it (F.M.P); mario.roggini@uniroma1.it (M.R.); gianmarcoandreoli@gmail.com (G.A.); m.davanzo@policlinicoumberto1.it (M.D.); 2 Institute of Translational Pharmacology, National Research Council, 00133 Rome, Italy; claudio.chiesa@ift.cnr.it

**Keywords:** NAFLD, subclinical atherosclerosis, cardiac structural and functional abnormalities, hypertension, type 2 diabetes, decreased bone mineral density, renal dysfunction, obstructive sleep apnea, polycystic ovary syndrome, children and adolescents

## Abstract

Background: There is growing evidence that non-alcoholic fatty liver disease (NAFLD) is a disease affecting not only the liver but also extrahepatic organs. Aim: To investigate whether in youths NAFLD is associated with extrahepatic complications such as subclinical atherosclerosis, cardiac abnormalities, hypertension, type 2 diabetes, decreased bone mineral density, renal dysfunction, obstructive sleep apnea, and polycystic ovary syndrome. Methods: We systematically reviewed PubMed; Scopus; Embase; and the Cochrane Library databases up to 28 February 2019 and assessed the quality of studies using the Newcastle-Ottawa Scale. Results: Thirty-five articles were selected for this systematic review: fifteen (4627 participants) evaluated the association of NAFLD with subclinical atherosclerosis; four (969 participants) with cardiac abnormalities; two (550 participants) with hypertension; four (1328 participants) with diabetes; six (523 participants) with low bone mineral density; two (865 participants) with renal dysfunction; one with obstructive sleep apnea; and one with polycystic ovary syndrome. Most studies found that youths with NAFLD have increased features of subclinical atherosclerosis; as well as of cardiac alterations. Limited data were available to endorse a solid estimate of the prevalence of diabetes; low mineral density and renal dysfunction in the pediatric NAFLD population. Conclusion: NAFLD-related intermediate CVD outcomes can occur and be detected early in young populations.

## 1. Introduction

**Figure 1:**
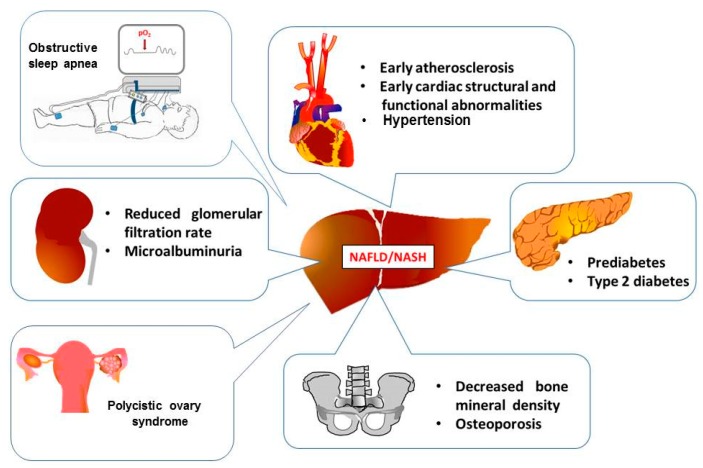
Extra-hepatic organ damage in youths with NAFLD.

Concurrent with the increasing prevalence of obesity, nonalcoholic fatty liver disease (NAFLD) has become a major cause of chronic liver disease in childhood [1]. NAFLD is defined as the presence of fat in the liver either on imaging or on liver histology after the exclusion of secondary causes of fat accumulation such as significant alcohol consumption, use of steatogenic medications, infections, or genetic/metabolic disorders.NAFLD encompasses a broad spectrum of liver disorders ranging fromsimple steatosis to nonalcoholic steatohepatitis (NASH). NAFLD can progress to advanced liver disease, cirrhosis and hepatocellular carcinoma and indication for liver transplantation [2]. Even though NAFLD is associated with increased liver-related mortality and morbidity, cardiovascular mortality is the main cause of death in NAFLD patients [3]. This has led to a growing awareness that NAFLD, as part of a spectrum of multiple-organ conditions termed metabolic syndrome (MetS), may be associated with extrahepatic complications, in particular type 2 diabetes, hypertension and cardiovascular disease (CVD), and chronic kidney disease (CKD) [4,5,6]. Emerging data also suggest NAFLD may be a risk factor for decreased bone mineral density (BMD) and osteoporosis, and colonic adenoma/cancer in adult patients [7,8,9]. Also, in youths, there is increasing evidence that NAFLD may be associated with extrahepatic disorders such as CVD, type 2 diabetes, decreased BMD, renal dysfunction, obstructive sleep apnea (OSA), and polycystic ovary syndrome (PCOS) (Figure 1) [10,11,12,13,14,15,16,17,18,19,20,21,22]. Thus, we here report the results of a systematic review of studies that have focused on the association of these extrahepatic complications with pediatric NAFLD. In view of the clinical impact and cost-effectiveness of the disease, elucidation of the extent of the potential adverse effects of NAFLD in children and adolescents could have implications for the prevention of this frequent disease through more rigorous surveillance and early treatment interventions.

## 2. Methods

### 2.1. Search Criteria

This systematic review was based on the Preferred reporting items for systematic review and meta-analysis protocols (PRISMA-P) checklist [23]. Relevant articles for the topics of interestwere determined by two independent reviewers (LP and FMP) by searching PubMed, Scopus, Embase, and the Cochrane Library databases up to 28 February 2019. The search was limited to children and adolescents (≤18 years). Search terms were nonalcoholic fatty liver disease, nonalcoholic steatohepatitis, hepatic steatosis in combination with children or adolescents; (a) subclinical atherosclerosis, intima-media thickness, arterial stiffness, flow-mediated dilation, pulse wave velocity; (b) left ventricular (LV) hypertrophy, LV dysfunction; (c) hypertension, elevated blood pressure; (d) type 2 diabetes, prediabetes, impaired glucose metabolism; (e) bone mineral density, osteoporosis; (f) chronic kidney disease, renal function, albuminuria; (g) obstructive sleep apnea, sleep disorders breathing; and (h) polycystic ovary syndrome. 

Additional relevant studies were found by looking at the references of articles identified by the above electronic databases.

### 2.2. Selection of Studies

Study selection was performed by two independent reviewers (LP and FMP) using inclusion and exclusion criteria. Inclusion criteria were: (1) articles published in English; and (2) observational (cohort or case-control) studies that compared the prevalence of cardiovascular, metabolic, renal and bone outcomes between patients with and without NAFLD. Yet, studies were eligible if NAFLD diagnosis was established by liver histology, or imaging evaluation, after exclusion of secondary causes of hepatic steatosis. 

Conference abstracts, theses, case reports, reviews, commentaries and editorials were excluded from the study. Studies with duplicate or overlapping protocols or research questions were excluded. Additional objectives of exclusion criteria were: (1) basic research studies; (2) studies where NAFLD diagnosis was only based on abnormal serum levels of liver enzymes; (3) studies without available outcome data of interest; and (4) studies lacking appropriateness for the topic. 

### 2.3. Data Extraction and Quality Assessment

Data extraction involved two independent reviewers (LP and CC) who collected data on specific points like study authors, title, date of publication; study design, and inclusion and exclusion criteria; study population including demographic and clinical features of cases and controls; diagnostic methods and study outcomes.

Finally, the quality of eligible articles was evaluated independently by two reviewers (LP and CC) on the basis of the Newcastle-Ottawa Scale (NOS) for cohort and cross-sectional studies [24]. Any disagreements were resolved by discussion or a third reviewer (FMP). 

## 3. Results

### 3.1. Characteristics of Included Studies and Participants

**Figure 2:**
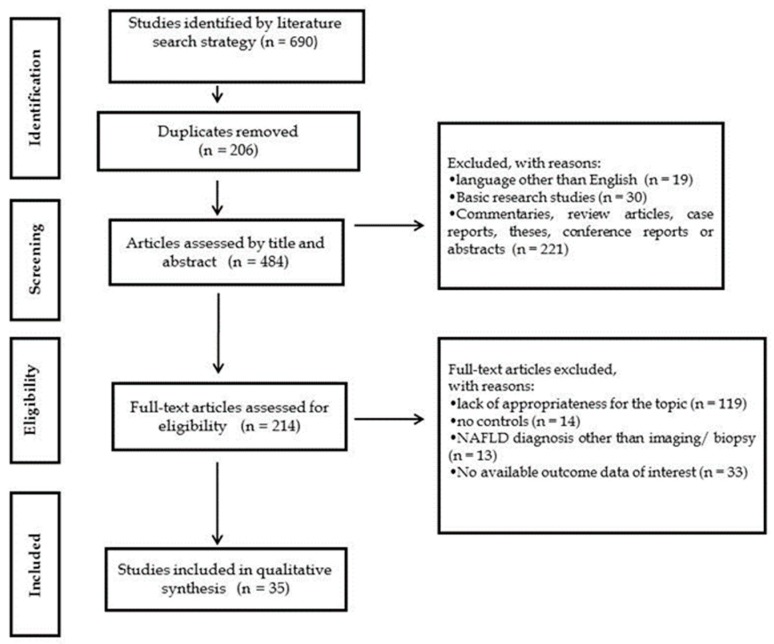
Flow diagram of the study selection process.

The main characteristics of the eligible studies are displayed in Table 1, Table 2, Table 3, Table 4, Table 5 and Table 6. The present systematic review included a total of thirty-five studies (Figure 2), of which fifteen (4627 participants) evaluated the association of NAFLD with subclinical atherosclerosis [17,25,26,27,28,29,30,31,32,33,34,35,36,37,38], four (969 participants) with cardiac structure and function abnormalities [32,33,39,40], two (550 participants) with hypertension [17,41], four (1328 participants) with type 2 diabetes [16,42,43,44], six (523 participants) with low BMD [14,15,45,46,47,48], two (865 participants) with renal dysfunction [13,49], one with OSA [19], and one with PCOS [21]. The majority of the studies were from Europe. 

### 3.2. Relationship between NAFLD and Atherosclerosis

Of the 15 studies providing data on subclinical atherosclerosis, ten were evaluated by carotid intima-media thickness (cIMT) [25,26,27,28,30,31,32,33,37,38], two by both cIMT and brachial artery flow mediated dilation (FMD) [17,35], one by cIMT and arterial stiffness [36], one by FMD alone [29], and one by arterial stiffness alone [34] (Table 1). The majority of the articles (10 of 13) evaluating cIMT supported the finding of elevated cIMT in children with NAFLD in comparison to those without liver involvement. On multivariate analysis after adjustment for potential confounders (including insulin resistance and other traditional risk factors), most studies demonstrated that presence of NAFLD remained associated with elevated cIMT. All ten studies used either abdominal ultrasound alone (*n* = 8) or abdominal ultrasound along with elevated liver enzymes [alanine aminotransferase (ALT) > 40 U/L] (*n* = 2) to identify NAFLD. In the remaining three studies defining NAFLD on the basis of liver ultrasound, magnetic resonance spectroscopy (MRS), and liver biopsy, respectively, the association between elevated cIMT and NAFLD was not found. The subgroup of children with biopsy-diagnosed NAFLD showed that in those with NAFLD cIMT was significantly increased on the left common carotid artery, though cIMT values overlapped substantially between patients and controls [27]. 

Three studies employed FMD as a surrogate marker of subclinical atherosclerosis. The first one involving 400 children and adolescents found reduced FMD in those with NAFLD when exposed to ischemia [17]. Using logistic regression analysis, after adjustment for demographic characteristics and MetS, the presence of NAFLD was shown to be an independent predictor of having low percentage FMD. The remaining two found no relationship between FMD and NAFLD. Weghuber et al. [29] who evaluated FMD in a very small cohort of obese children with (*n* = 14) or without (*n* = 14) MRS-diagnosed NAFLD, found a similar FMD response between the two groups. Torun et al. [35] who assessed both cIMT and FMD, found that obese children with hepatosteatosis exhibited increased cIMT versus the non-hepatosteatosis obese group as well as the healthy controls. Though FMD decreased as the grade of hepatosteatosis increased, it did not reach a significant level.

Finally, two studies analyzed the relation of NAFLD with carotid artery stiffness. The former study [34] evaluating in 964 Australian adolescents the relationship between ultrasound-diagnosed NAFLD and arterial stiffness, demonstrated that NAFLD is associated with raised arterial stiffness only in subjects belonging to the “high metabolic risk” group (as based on systolic arterial pressure, homeostasis model assessment of insulin resistance, triglycerides, and body mass index (BMI)), therefore suggesting that arterial stiffness related to NAFLD is predicated on the presence of an adverse metabolic profile in adolescents. The latter one evaluating both cIMT and arterial stiffness in 78 obese children with (*n* = 41) or without (*n* = 37) NAFLD, found no difference in cIMT and arterial stiffness between the two groups [36].

### 3.3. Relationship between NAFLD and Cardiac Abnormalities

In Table 2 are presented the four studies evaluating in childhood the association between NAFLD and abnormalities in cardiac structure and function [32,33,39,40]. In three articles [32,33,40], M-mode echocardiography, pulsed Doppler and tissue Doppler echocardiography were used to measure LV structure and function. In this respect, a total of 688 obese children (of whom 244 with and 444 without NAFLD) and 236 age- and sex-matched healthy controls were included. NAFLD was identified by ultrasonography in one study, by ultrasonography and elevated serum ALT in one study, and by magnetic resonance imaging (MRI) (and liver biopsy in a subgroup) in one study.LV mass indexed to height^2.7^ or to body surface area, early mitral velocity (*E*)/late mitral velocity (*A*) ratio, the early annular diastolic tissue velocity (*e*′), and *E*/*e*′ ratio were the outcomes most commonly reported. Increased LV mass in NAFLD youths in comparison to patients without NAFLD was reported in 2 of the 3 studies, while in the third one only increased interventricular septum thickness was found. A significant association between NAFLD and diastolic cardiac dysfunction was observed in all three studies. Notably, among children with biopsy-diagnosed NAFLD, those with severe liver histologic features presented worse diagnostic LV dysfunction than those with milder liver injury.

Using integrated backscatter ultrasonography and speckle tracking echocardiography, the fourth article measured LV geometry and function in 3 groups of age-, sex-, and Tanner stage-matched adolescents having been classified, respectively, as normal weight (*n* = 14), obese with (*n* = 15) or without MRS-diagnosed NAFLD [39]. Compared to lean adolescents, obese subjects presented decreased systolic and early diastolic strain rates. Compared to obese adolescents without NAFLD, in those with NAFLD early diastolic longitudinal strain rates were significantly decreased.

### 3.4. Relationship between NAFLD and Hypertension

Two studies have specifically addressed the prevalence of elevated blood pressure in pediatric patients with NAFLD as compared to those without NAFLD (Table 2). In the first case-control study including 150 overweight children with biopsy-proven NAFLD and 150 overweight subjects without NAFLD matched in age, gender, and severity of obesity, Schwimmer et al. [41] found that children with NAFLD had a significantly higher frequency of elevated blood pressure (32% vs. 16%; *P* = 0.001). The second study involving 400 children and adolescents found that children with NAFLD had a prevalence of hypertension of 61% as compared to 36.6% in those without NAFLD (*P* = 0.0001) [17].

There are additional studies evaluating the prevalence of hypertension in pediatric patients with NAFLD, but without comparing data in subjects without NAFLD. In a cohort of 120 children and adolescents (aged 3–18 years) with biopsy-proven NAFLD, Manco et al. [50] reported a 40% prevalence of hypertension. In a longitudinal study involving 382 children and adolescents (aged 2–17 years) with biopsy-proven NAFLD, prevalence of high blood pressure at baseline was 35.8% and prevalence of persistent high blood pressure was 21.4% at 48-week follow-up [51]. Patients with hypertension had more severe grades of steatosis than those without hypertension.

### 3.5. Relationship between NAFLD andType 2 Diabetes (Glucose Intolerance)

Four observational cohort studies compared the prevalence of diabetes in patients with and without NAFLD (Table 3) [16,42,43,44]. The first report investigating 571 obese children in regards to the relationship between ultrasound-diagnosed NAFLD and glucose metabolism, showed that the prevalence of prediabetes/diabetes was significantly increased in those with NAFLD [42]. The second study including 451 pubertal obese children also found that the prevalence of type 2 diabetes was significantly increased in patients with NAFLD compared to those with no liver involvement [43]. The third study involving obese adolescents who underwent bariatric surgery demonstrated that in subjects without NAFLD, the prevalence of type 2 diabetes was 6.6% and increased progressively to 8.8% in NAFLD (not-NASH), to 23.8% in borderline NASH, and to 66.7% in NASH [44]. Finally, in an observational study including 158 overweight/obese subjects (mean age, 12 years) with or without MRI-diagnosed NAFLD, a higher prevalence of prediabetes was found in those with NAFLD [16].

Notably, other studies have reported the prevalence of type 2 diabetes (range between 2% and 14%) in youths with NAFLD, but without comparing data in subjects without NAFLD [18,50,52,53]. The majority of them were also limited by small sample size. The one involving a large sample (*n* = 675) of children with biopsy-confirmed NAFLD reported a prevalence of prediabetes and diabetes of 23.4% (158 of 675) and 6.5% (44 of 675), respectively. Prevalence of NASH was higher in patients with type 2 diabetes (43.2%) than in those with prediabetes (34.2%) or normal glucose (22%). Among patients with type 2 diabetes, 75% (33 of 44) had a preexisting clinical diagnosis of the disease. Importantly, HbA1C, but not oral glucose tolerance test (OGTT), was used in addition to fasting glucose for both diagnoses of prediabetes and diabetes [18]. Finally, in a longitudinal but retrospective cohort study involving 66 children with NAFLD (mean age 13.9 ± 3.9 years), Feldstein et al. [54] showed that at presentation impairment of fasting glucose was a common finding (12.1%), and that over a follow-up period spanning 4 to 11 years a significant percentage of NAFLD children (6.1%) developed overt diabetes.

### 3.6. Relationship between NAFLD and Bone Mineral Density

In Table 4 are presented the six studies (five observational and one longitudinal) [14,15,45,46,47,48] evaluating the relationship between NAFLD and BMD.NAFLD diagnosis was based on ultrasonography (three studies), MRI (one study), liver biopsy alone (one study) or in combination with MRI (one study). In all six articles, total body or lumbar spine BMD *z* scores were measured by dual-energy X-ray absorptiometry in children and adolescents with NAFLD and without hepatic involvement. There were significantly decreased BMD *z* scores in youths with NAFLD compared to controls in 4 of the six studies involving a total of 391 youths [14,15,45,48]. The remaining two studies involving a total of 80 obese children with NAFLD and 54 controls, found no association between NAFLD and BMD [46,47]. Interestingly, both studies were performed in non-European (Brazilian and South-Korean) subjects. Among the selected articles, two of them providing data on the relationship between severity of liver histology and BMD [14,15], showed significantly decreased BMD *z* scores in patients with NASH than in those without NASH. 

Two additional cross-sectional studies have evaluated BMD in youths with NAFLD, but without comparing data in subjects without NAFLD [55,56]. Both studies demonstrated significantly lower total body BMD *z* scores in adolescents with NASH compared to those with no-NASH.

### 3.7. Relationship between NAFLD and Renal Function

Two studies provided data on renal function in youths with NAFLD (Table 5) [13,49]. In the former study, urinary albumin excretion and creatinine clearance were not different between children with biopsy-proven NAFLD (*n* = 80) and normal-weight subjects (matched for age and sex) (*n* = 59) who were chosen among youths admitted to the hospital for minor surgical intervention over the same period of time [49]. However, a significant, but weak, inverse relationship between insulin sensitivity and creatinine clearance was observed in NAFLD children. There were no differences in renal function between NAFLD patients presenting with or without MetS, low or normal high-density lipoprotein cholesterol, and different degrees of histopathologic liver features. Only subjects with high blood pressure had increased values of albuminuria. The latter study involving 596 overweight/obese patients of whom 268 with and 328 without MRI-diagnosed NAFLD, and 130 healthy lean subjects reported a greater prevalence of low (less than 90 mL/min/1.73 m^2^) estimated glomerular filtration rate (eGFR) in children with NAFLD than in those without NAFLD, as well as in healthy controls [13]. Likewise, subjects with NAFLD had a higher prevalence of microalbuminuria in comparison to those without NAFLD as well as healthy controls. On multiple logistic regression analysis, NAFLD was associated with an increased risk of reduced eGFR and/or microalbuminuria (odds ratio, 2.54 (confidence interval, 1.16–5.57); *P* < 0.05), after adjustment for anthropometric and clinical parameters.

### 3.8. Relationship between NAFLD and Obstructive Sleep Apnea

In children and adolescents, few studies have investigated the relationship between OSA and NAFLD, but only one fulfilled the inclusion criteria for study selection (Table 6). In this respect, Verhulst et al. [19] reported a relationship between sleep-disordered breathing and suspected fatty liver disease (defined as elevated serum aminotransferase levels and/or hyperechoic liver on ultrasound) in overweight children and adolescents. 

Other studies have investigated the association between NAFLD and OSA, but were excluded for inadequate definition of NAFLD (based solely by liver enzymes elevation) or lack of non-NAFLD controls. In this respect, Kheirandish-Gozal et al. [20] evaluated the prevalence of elevated liver enzymes in 518 habitually snoring children. Of the 343 children with confirmed OSA, 44 (12.8%) had elevated liver enzymes compared to only five children among the 175 subjects without OSA (2.8%; *P* < 0.0001). Effective treatment of OSA resulted in improved liver function test in the majority of these patients. In two additional studies [57,58], polysomnographic evaluation of youth with biopsy-proven NAFLD demonstrated a prevalence of OSA of about 60%. Also, the presence and severity of OSA was associated with the severity of liver histology, independently of whole-body/ abdominal obesity, metabolic syndrome, and insulin resistance. 

### 3.9. Relationship between NAFLD and Polycystic Ovary Syndrome

In adolescents, only one study has investigated the relationship between PCOS and NAFLD, fulfilling the inclusion criteria for study selection. Community-based adolescents from the Raine Cohort participated in assessments for NAFLD and PCOS [21]. Among the 199 girls who attended both assessments, PCOS was diagnosed in 16.1% and NAFLD in 18.6%. NAFLD was more prevalent in girls with PCOS than girls without PCOS (37.5% vs. 15.1%; *P* = 0.003). Adolescents with NAFLD and PCOS had greater adiposity, inflammatory markers, and serum androgens than those with NAFLD but without PCOS.

Other studies have investigated the association between NAFLD and PCOS, but were excluded for inadequate definition of NAFLD (based solely by liver enzymes elevation) or lack of controls. In a small sample of 30 Tanner stage V obese girls with PCOS, Michaliszyn et al. [22] found that the prevalence of NAFLD (as assessed by computed tomography) was 6.7%. In a retrospective study involving 39 obese adolescent females with PCOS, Barfield et al. [59] demonstrated that 15.4% of them had elevated levels of aminotransferases.

ALT = alanine aminotransferase; AST = aspartate aminotransferase; BMI = body mass index; BMI-SDS = BMI-standard deviation score;BP = blood pressure; HDL = high-density lipoprotein cholesterol; HOMA-IR = homeostasis model of insulin resistance; LDL = low-density lipoprotein cholesterol; LV = left ventricular; MRI = magnetic resonance imaging; NAFLD = nonalcoholic fatty liver disease; MRS = magnetic resonance spectroscopy; TC = total cholesterol; TG = triglycerides; WBISI = whole body insulin sensitivity.

## 4. Discussion

The research to date illustrates that NAFLD is associated with various metabolic and CVD comorbidities in adults. Although studies in the field are limited in regards to children and adolescents, the ones we analyzed revealed that NAFLD-related intermediate metabolic and CVD outcomes can occur and be detected early in young populations. This emphasizes the problem is not only one of cardio-metabolic risk in the long run but also one requiring prompt attention to target and prevent unnoticed organ damage in the early stages, as well as to develop proper follow-up strategies in children and adolescents with NAFLD.

### 4.1. NAFLD and Cardiovascular Abnormalities

Atherogenesis begins in early life. It has been reported among children as young as 2 years of age, in association with well-known components of MetS, such BMI, dyslipidemia, and systolic and diastolic blood pressure [60]. 

In this systematic review, we revealed a close link in youths between NAFLD, a novel component of MetS, and clinical parameters of subclinical atherosclerosis like increased cIMT, arterial stiffness, and endothelial dysfunction, though the strength of the association was mainly investigated by the assessment of cIMT. Our present results confirm and expand on the findings of a previous systematic review and meta-analysis including five pediatric studies, predominantly involving obese children [61]. The meta-analysis demonstrated that youths with NAFLD had a significantly increased cIMT in comparison with subjects with no-NAFLD according to both random [standardized mean difference (SMD) = 1.083, 95% confidence interval: 0.457–1.709; *P* = 0.001, I2 = 0) and fixed effects models (SMD = 0.995, 95% confidence interval: 0.840–1.150; *P* < 0.001, I2 = 93%). Of note, the percent increase in cIMT was estimated to be 14.98% among patients with NAFLD as compared to cIMT values in subjects without NAFLD. Findings from our systematic review as well as from previous meta-analysis [61] assume considerable importance because early-onset atherosclerosis in pediatric patients with NAFLD may be responsible of a greater burden of CVD in adults. Carotid atherosclerosis may indicate the presence of atherosclerotic lesions in other arteries and may serve as a signal of systemic disease. As a matter of fact, elevated cIMT has been correlated with an increased risk for cardiovascular, cerebrovascular, and peripheral vascular disease [62,63]. Therefore, cIMT as evaluated by B-mode ultrasonography, may represent a useful radiologic modality to demonstrate subclinical atherosclerosis and its progression.

Many studies suggest that NAFLD is associated with an increased risk of CVD, independently of cardiometabolic risk factors [3]. Considering many children with NAFLD may or may not have additional metabolic or clinical risk factors, future studies should ascertain whether the assessment of vascular structure and function among children with NAFLD may be a sensitive, effective clinical indicator of atherosclerosis in those at low as well as high metabolic risk.

Contemporary research suggests that NAFLD may also be a risk factor for subclinical abnormalities in cardiac structure and function [64]. Accordingly, our systematic review indicates that youths with NAFLD have increased hallmarks of diastolic LV dysfunction as well as of LV hypertrophy. Diastolic cardiac dysfunction is recognized as a major contributor to the development of heart failure. However, it is often diagnosed rather late since the onset of heart failure with preserved systolic function is asymptomatic [65,66]. Application of new imaging markers including tissue velocity (e’ and E/e’) and speckle tracking echocardiography may provide evidence of subtle cardiac dysfunction. LV hypertrophy, a form of end organ damage, is often reported to occur in patients with hypertension as well as with obesity [67]. This abnormality of cardiac geometry, though commonly asymptomatic in children, has been associated in adults with complications such as ventricular arrhythmias, myocardial infarction, cerebrovascular events, congestive heart failure, and death [68]. Indeed, from our systematic review, LV hypertrophy was not observed in all selected studies. Nonetheless, it is important to point out that LV diastolic dysfunction is recognized to be associated with the development of LV hypertrophy, and that LV diastolic dysfunction may precede the development of LV hypertrophy [69].

The effects of NAFLD on cardiac abnormalities has been investigated in few studies always based on statistical adjustments. However, confirmation of the role of NAFLD *per se* (isolated from its well-known associated metabolic risk factors) is needed in large cross-sectional and longitudinal studies.

In the past decade, genome-wide association studies have revealed that several single nucleotide polymorphisms (SNPs) are associated with a high risk for NAFLD development and progression. Among them, the Patatin-like phospholipase domain-containing 3 (PNPLA3) gene variant I148M showed a strong relationship not only with hepatic fat content and increased serum liver enzymes but also with increased risk of NASH and fibrosis progression [70,71,72]. The mutation causes an intracellular impairment in lipid droplet remodeling and very-low density lipoproteins (VLDL) secretion in hepatocyte without apparently affecting body mass, serum lipid levels and insulin resistance. Recent studies have found that I148M mutation was associated with increased vascular damage in a high-risk Mexican population with NAFLD [73], but not in Chinese subjects [74]. The transmembrane 6 superfamily member 2 (TM6SF2) gene variant E167K was also associated with NAFLD, and it has a relationship with cardiovascular disease. The TM6SF2 E167K variant increases liver fat content, inflammation and fibrosis by interfering with the efflux of fat from the hepatocyte (i.e., lipidation and secretion of apolipoprotein B during VLDL secretion). As a consequence, subjects carrying the mutations have high hepatic fat content, lower circulating levels of apolipoprotein B-rich lipoproteins such as triglycerides and low-density lipoprotein cholesterol, and thus a lower CVD risk. Dongiovanni et al. [75] in a large cohort of European descent found that the TM6SF2 E167K variant was associated with NASH and advanced liver fibrosis but it was protective against carotid plaques, a strong predictor of atherosclerosis burden and CV risk. 

Though preclinical atherosclerosis is a dynamic process, the analysis of our results shows that none of the selected studies have evaluated the reversibility of clinical parameters of subclinical atherosclerosis after lifestyle interventions. Nonetheless, it may be of great interest that previous studies in obese children including those with NAFLD have shown that lifestyle interventions can slow or even regress the progression of atherosclerosis [76,77,78,79,80]. 

### 4.2. NAFLD and Type 2 Diabetes

NAFLD is deeply linked to an alteration ofglucose metabolism [4,6,81,82]. In adults, NAFLD has been often reported to precede type 2 diabetes, being involved in its developmentby means of increasing hepatic as well as whole body insulin resistance driving alteration in glucose metabolism [83,84]. On the other hand, type 2 diabetes is featured by whole-body insulin resistance, elevated levels of insulinand a degree of resistance to the effect of gut hormones [82]. This is responsible for enhanced *de novo* lipogenesis together with decreased fat oxidation in the liver, thus leading toincreased hepatic fat content, and resulting in a vicious cycle ultimately leading to an increased risk for NASH, and all-cause as well as liver-related mortality [85,86]. 

It may be therefore of great interest the recent meta-analysis by Mantovani et al. [83] who demonstrated an enhanced risk of incident type 2 diabetes following an imaging-based diagnosis of NAFLD. This meta-analysis involving 19 observational studies with aggregate data on 296,439 adult subjects (of whom 30.1% with NAFLD) and nearly 16,000 cases of incident diabetes, showed that the presence of NAFLD conferred hazard ratio of 2.2 for incident diabetes over a median follow-up period of 5 years. Importantly, the meta-analysis also demonstrated that the risk of incident diabetes was higher in some ethnic groups (predominantly in Japanese subjects), in patients with more severe forms of NAFLD, and in studies with longer follow-up. 

Limited data are available on the prevalence of prediabetes or type 2 diabetes in youths affected by NAFLD [16,18,42,43,44,50,52,53]. There is one longitudinal but retrospective cohort study in youths with NAFLD who were identified on the basis of diagnostic codes of the International Classification of Diseases for fatty liver, hepatic steatosis or steatohepatitis [54]. As OGTT was not carried out, the authors utilized a fasting glucose level of at least 100 mg/dl to replace impaired glucose tolerance. At presentation, of the 66 study patients eight (12.1%) presented an impaired fasting glucose while two (3.0%) were diagnosed as having diabetes. At the time of the last follow-up (mean 6.4 ± 4.5 years; range 0.05 to 20), four (6.1%) children with baseline normal fasting glucose developed overt diabetes 4, 6, 7, and 11 years, respectively, after the diagnosis of NAFLD. Studies also indicate that NAFLD strongly influences the metabolic parameters in the longitudinal setting. In particular, Kim et al. [87] showed in a sample of 76 obese children that glucose (fasting and 2-h blood glucose) as well as insulin sensitivity indices at a mean follow-up of 1.9 years were significantly related to baseline liver fat content. Notably, over the follow-up, the authors observed that beta-cell function significantly improved in children with low versus those with high hepatic fat fraction. Adding to this, the few cross-sectional studies available in the pediatric literature demonstrate that the prevalence of diabetes in overweight/obese children and adolescents with NAFLD is higher when compared to that in overweight/obese subjects without liver involvement [16,42,43,44]. 

So far, sample sizes are too small to endorse a solid estimate of the prevalence of type 2 diabetes in the pediatric NAFLD population. Since morbid complications are already present in overt type 2 diabetes, it becomes crucial to identify NAFLD children with prediabetes, namely those with impaired glucose tolerance. It is well-known that insulin resistance is a key feature of NAFLD, in addition to obesity associated insulin resistance. Unfortunately, though OGTT can reveal insulin resistance, it is not routinely applied in NAFLD patients because of the high cost and inconvenience. Thus, it is an urgent issue to determine the indications for OGTT in NAFLD subjects. 

Future longitudinal studies of NAFLD subjects with normal OGTT as well as normal fasting glucose may give us more knowledge about the natural course of NAFLD. 

### 4.3. NAFLD and Bone Mineral Density

A very recent meta-analysis showed that youths with imaging-or biopsy-diagnosed NAFLD had decreased total body or lumbar BMD *z* scores compared to children without NAFLD (pooled weighted mean difference: −0.48, 95% confidence interval −0.74 to −0.21; *I*2 = 55.5%) [88]. Furthermore, a significant difference in total body BMD *z* scores between children with and without histologically proved NASH was reported (pooled weighted mean difference: −0.27, 95% confidence interval −0.40 to −0.13; *I*2 = 0%). Of note, in most of the included studies the aforementioned pooled weighted mean difference in BMD *z* scores was independent of age, gender, race/ethnicity and BMI.

From our systematic review, of the 6 eligible studies, 4 found a significant association between NAFLD and reduced BMD, while 2 did not. Possible reasons accounting for such discrepant results include differences in study design, demographic and ethnic features of study populations, lifestyle habits, severity and duration of obesity as well as of NAFLD, and adjustments for established risk factors for BMD as well as for NAFLD. These findings argue for planning careful monitoring and evaluation of BMD in large, worldwide pediatric cohorts with NAFLD. The potential contribution of NAFLD *per se* to the development and progression of reduced BMD warrants further studies.

### 4.4. NAFLD and Renal Function

Currently, NAFLD is considered as an additional component of MetS, whose chief underlying cardiometabolic risk factors are abdominal obesity and insulin resistance [89,90]. In addition to represent a central feature in the development of NAFLD, insulin resistance is also a predictor of incident CKD [91,92]. Furthermore, both atherogenic dyslipidemia and type 2 diabetes are well known risk factors for CKD [93,94]. As such, NAFLD has been suggested to play a potential pathogenic role in the occurrence of CKD. The results of a recent meta-analysis have delineated that adult patients with NAFLD have an increased risk of CKD, and that the increased risk and severity of CKD in these patients are related to the degree of hepatic disease. These associations were independent of well-established risk factors for CKD, including total body/abdominal obesity and insulin resistance [95]. In children, the data are scanty. The study by Pacifico et al. [13] including a large cohort of children and adolescents with an extensive analysis of metabolic parameters, suggests that obese youths with NAFLD are at risk for early renal dysfunction. Awareness of this alteration in youths may be of crucial importance since measures to reverse the disease process are most likely to be efficacious if applied in the early stages. 

### 4.5. NAFLD and Obstructive Sleep Apnea

OSA, characterized by recurrent partial or complete upper airway obstruction during sleep, affectsup to 6% of the general pediatric population and up to 78% of obese children [96,97]. Symptoms of OSA include daytime sleepiness, poor school performance, and snoring, though many children may be asymptomatic [98].Affected patients experience repeated episodes of nocturnal hypoxia alternating with normoxia (chronic intermittent hypoxemia), resembling the pathophysiological mechanisms involved in ischemia/reperfusion tissue injury [99,100]. Emerging research suggests that obesity-related OSA and intermittent nocturnal hypoxia are associated with NAFLD disease severity and progression [57,58]. Pediatric patients with NAFLD and OSA/hypoxia have been shown to have more advanced liver disease and fibrosis than those without OSA/hypoxia. OSA/ nocturnal hypoxia may trigger oxidative stress, through reactive oxygen species generation, that promotes progression of NAFLD [101]. Interestingly, a very recent pilot study involving nine adolescents with NAFLD provided strong evidence that treatment of OSA /nocturnal hypoxia with continuous positive airway pressure may reverse parameters of liver injury and reduce oxidative stress [102].

### 4.6. NAFLD and Polycystic Ovary Syndrome

PCOS is a common endocrine disorder in females of reproductive age, characterized by menstrual irregularities and clinical and/or biochemical hyperandrogenism in the presence or absence of polycystic ovaries, with most patients being overweight or obese [103]. Adolescents with PCOS often have MetS, insulin resistance and impaired glucose tolerance. Since PCOS and NAFLD share common metabolic abnormalities, it is not surprising that NAFLD is highly prevalent in adolescent females with PCOS. 

### 4.7. Limitations

Our study presents some limitations. First, any systematic review is conducted in a retrospective manner that may be influenced by the methodological strength of selected articles, exhaustive search procedures, and publication bias. To lessen such restrains, we used a rigorous search methodology using explicit inclusion and exclusion criteria for the articles included in the review. Second, all but one of the 35 studies available for this meta-analysis were cross-sectional. Thus, it is difficult to drive causal or temporal relationships between NAFLD and outcomes of interest. Third, the included studies were characterized by a relatively small sample size as well as a medium-high risk of bias (according to the NOS scale). Fourth, most selected articles reported incomplete risk-adjustments. Finally, in the majority of the studies NAFLD was diagnosed by ultrasound which is qualitative and therefore subjective. However, as per the consensus practice guidelines by the North American and European Society for Pediatric Gastroenterology, Hepatology and Nutrition, liver ultrasound is recommended as the first-line investigation for the diagnosis of NAFLD, without a need for a liver biopsy, an invasive procedure associated with inherent risks [104].

## 5. Conclusions

As the hepatic manifestation of MetS, NAFLD has shown clear associations with CVD, type 2 diabetes, and CKD. On the basis of available evidence from cross-sectional studies, youths with NAFLD are more likely to be at risk for early atherosclerotic changes and cardiac alterations, and metabolic derangement.Prospective longitudinal studies are needed to establishthe extent to which pediatric NAFLD and its severity may impact on long-term cardiovascular outcomes in the communitypopulation. In particular, these studies may elucidate whether the increased risk of atherosclerotic changes and cardiac alterations might reflect the cluster of underlying metabolic risk factors, or whether NAFLD *per se*, primarily NASH, might confer a risk of adverse cardiovascular outcome above and beyond that associated with the single components of MetS. Future long-term studies are also needed to understand the temporal relationship between NAFLD and type 2 diabetes as well as to clarify the complex interplay of factors involved in the development of bone and renal complications. In that vein, of greatest interest is the study by Allen et al. [105] suggesting that NAFLD is a complex and heterogeneous disease that can promote or accelerate the progression of CV risk factors. The authors also showed that the impact of NAFLD on incident type 2 diabetes, hypertension or dyslipidemia decreases as the subject progresses to higher dysmetabolic state. Thus, clinical programs for early diagnosis of NAFLD and related interventionshould be introduced before the onset of a high dysmetabolic burden.

## Figures and Tables

**Table 1:** Study characteristics for nonalcoholic fatty liver disease and atherosclerosis.

Author/Country/ Year	Study Design	Study population (NAFLD vs. Non-NAFLD)	Diagnosis of NAFLD	Outcome Measures	Results	Adjustment Considered	NOS
Pacifico et al. /Italy/ 2008 [25]	Cross-sectional	Hospital-based cohort of obese children with (*n* = 29), and without NAFLD (*n* = 33); healthy lean controls undergoing checkups in hospital clinic (*n* = 30)	Liver ultrasound	cIMT [mean (95% CI), mm]	NAFLD vs. non-NAFLD and controls: 0.54 (0.50–0.58) vs. 0.45 (0.42–0.48) and 0.39 (0.35–0.43); *P* < 0.01 and *P* < 0.0005, respectively	Age, gender, Tanner stage, and cardiovascular risk factors	8
Demircioglu et al. /Turkey/2008 [26]	Case-control	Hospital-based cohort of obese children (*n* = 80) with mild (group 3, *n* = 32), moderate-severe NAFLD (group 4, *n* = 22), and without NAFLD (group 2, *n* = 26); and healthy children admitted to the hospital for medical control(group 1, *n* = 30) matched for age and gender	Liver ultrasound	cIMT [mean (SD), mm]	Groups 4, 3, 2, 1: Left CCA, 0.444 (0.083), 0.415 (0.065), 0.389 (0.06), 0.352 (0.054); *P* = 0.009	Age, gender, weight, ALT levels, TC, obesity, and grade of steatosis	8
Manco et al./Italy/ 2010 [27]	Case-control	Hospital-based cohort of overweight/obese children with (*n* = 31), and without (*n* = 49) NAFLD, matched for age, gender, and BMI	Liver biopsy	cIMT [median (IQR), mm ]	NAFLD vs. non-NAFLD: Right cIMT, 0.47 (0.07) vs. 0.48 (0.05); *P* = 0.659. Left cIMT, 0.49 (0.12) vs. 0.47 (0.05); *P* = 0.039	Age, gender, BMI, BP, and TG	8
Caserta et al./Italy/ 2010 [28]	Cross-sectional	Population-based cohort of randomly selected adolescents (*n* = 642) of whom 30.5% and 13.5% were, respectively, overweight and obese. Overall prevalence of NAFLD, 12.5%	Liver ultrasound	cIMT [mean (95% CI), mm]	NAFLD vs. non-NAFLD: 0.417 (0.409–0.425) vs. 0.395 (0.392–0.397); *P* < 0.001	BMI, WC, BP, HDL, ALT, GGT, CRP	8
Pacifico et al./Italy/ 2010 [17]	Cross-sectional	Hospital-based cohort of obese children with (*n* = 100), and without (*n* = 150) NAFLD; healthy lean controls recruited from schools (*n* = 150)	Liver ultrasound and elevated ALT	cIMT [mean (95% CI), mm] and FMD [mean (95% CI), %]	NAFLD vs. non-NAFLD and controls: cIMT, 0.47 (0.45–0.49) vs. 0.44 (0.43–0.46) and 0.40 (0.39–0.41); *P* < 0.0001 and *P* < 0.01, respectively; FMD, 6.7 (5.0–8.6) vs. 11.8 (10.1–13.7) and 15.0 (13.9–17.3); *P <* 0.01 and *P <* 0.001, respectively.	Age, gender, Tanner stage, and MetS components	8
Weghuber et al. /Austria/2011 [29]	Cross-sectional	Hospital-based cohort of obese children with (*n* = 14), and without (*n* = 14) NAFLD	MRS	FMD, [mean (SD), %]	NAFLD vs. non-NAFLD: 110.7 (9.0) vs. 108.6 (11.8); *P* = 0.41	BMI	8
Akin et al./Turkey/ 2013 [30]	Cross-sectional	Hospital-based cohort of obese children with (*n* = 56), and without (*n* = 101) NAFLD	Liver ultrasound	cIMT, [mean (95% CI), mm]	NAFLD vs. non-NAFLD: 0.48 (0.47–0.49) vs. 0.45 (0.44–0.45); *P* < 0.001	Age and gender	8
Gökçe et al./Turkey/ 2013 [31]	Cross-sectional	ospital-based cohort of obese childrenH with (*n* = 50), and without (*n* = 30) NAFLD; healthy lean controls (*n* = 30)	Liver ultrasound	cIMT, [mean (SD), mm]	NAFLD vs. non-NAFLD vs. control group: Right cIMT, 0.46 (0.21) vs. 0.35 (0.09) vs. 0.30 (0.13); *P* < 0.01 Left cIMT, 0.44 (0.09) vs. 0.35 (0.08) vs. 0.27 (0.04); *P* < 0.01.	Age, gender, BMI, BP, TG, HDL, IR, and MetS	8
Sert et al./Turkey/ 2013 [32]	Cross-sectional	Hospital-based cohort of obesechildren with (*n* = 97), and without (*n* = 83) NAFLD; healthy lean controls recruited from subjects seen in hospital for minor illnesses (*n* = 68)	Liver ultrasound and elevated ALT	cIMT, [mean (SD), mm]	NAFLD vs. non-NAFLD vs. Controls: 0.437 (0.028) vs. 0.383 (0.019) vs. 0.354 (0.009); *P* < 0.05 and *P* < 0.05, respectively	Age, waist –to- hip ratio, BP, HOMA-IR, TC, HDL, AST and ALT levels	7
Alp et al./Turkey/ 2013 [33]	Cross-sectional	Hospital-based cohort of obese children with moderate NAFLD (group 3, *n* = 26), mild (group 2, *n* = 67), and without NAFLD (group 1, *n* = 307); and healthy lean controls recruited from subjects seen in hospital for minor illnesses (group 1, *n* = 150), matched for age and gender	Liver ultrasound	cIMT, [mean (SD), cm]	Moderate NAFLD vs. mild NAFLD vs. non-NAFLD vs. controls: 0.10 (0.01) vs. 0.09 (0.01) vs. 0.09 (0.01) vs. 0.06 (0.01); *P* < 0.001	Anthropometric variables, total fat mass, HOMA-IR	8
Huang et al./Australia/ 2013 [34]	Cross-sectional	Population-based cohort of male adolescents (*n* = 509, of whom 55 with and 454 without NAFLD); and female adolescents (*n* = 455, of whom 77 with and 378 without NAFLD)	Liver ultrasound	PWV, [mean (SD), m/sec]	Non-NAFLD, low metabolic risk (MR) vs. NAFLD, low MR vs. non-NAFLD, high MR vs. NAFLD, high MR: males, 6.6 (0.7) vs. 6.7 (0.7) vs. 6.7 (0.6) vs. 6.9 (1.0); females, 6. 2 (0.7) vs. 6.3 (0.7) vs. 6.5 (0.7) vs. 6.4 (0.6). Males and females with both NAFLD and high MR had greater PWV compared to those who were disease- risk free [b, 0.20 (95% CI 0.01 to 0.38); *P* = 0.037]	Age, gender, smoking	8
Torun et al./Turkey/ 2014 [35]	Cross-sectional	Hospital-based cohort of obese children with mild (group 3, *n* = 47), moderate-severe NAFLD (group 4, *n* = 38), and without NAFLD (group 2, *n* = 24); healthy lean controls (group 1, *n* = 44)	Liver ultrasound	cIMT [median (IQR), mm] and FMD[median (IQR), mm]	Groups 4, 3, 2, 1: cIMT, 0.52 (0.10), 0.50 (0.10), 0.43 (0.06), 0.43 (0.09); *P* < 0.001 FMD, 0.055 (0.04), 0.060 (0.04), 0.065 (0.08), 0.060 (0.07); *P* = 0.392	ALT levels, BMI -SDS and grade of steatosis	7
Koot et al./The Netherlands/2015 [36]	Cross-sectional	Hospital-based cohort of obese children with (*n* = 41), and without NAFLD (*n* = 37)	MRS	cIMT [mean (SD), mm] and carotid stiffness [mean (SD)]	NAFLD and NAFLD plus elevated ALT vs. non- NAFLD: cIMT, 0.48 (0.06) and 0.48 (0.07) vs. 0.47 (0.06); *P* = 0.61 and 0.59, respectively. Carotid stiffness, 3.0 (0.81) and 2.90 (0.78) vs. 2.78 (0.50); *P* = 0.59 and 0.18, respectively	Age, gender, pubertal stage, BMI z score, WC, BP, LDL/HDL, IR	8
Daar et al./Turkey/ 2016 [37]	Cross-sectional	Hospital-based cohort of obese (*n* = 53) and age- and sex-matched non obese (*n* = 53) children; 81.1% of the obese (*n* = 43) and 7.5% of the non-obese group (*n* = 4) had NAFLD	Liver ultrasound	cIMT, [median (IQR), mm]	NAFLD vs. non-NAFLD: 0.95 (0.90–1.10) vs. 0.65 (0.60–0.80); *P* < 0.001	-	7
Rutigliano et al./ Italy/2017 [38]	Cross-sectional	Hospital-based cohort of obese children (*n* = 803 of whom 105 and 172 had MetS and NAFLD, respectively); control group recruited from children seen in hospital for functional bowel disorders (*n* = 63)	Liver ultrasound	cIMT, [mean (SD), mm]	HMR vs. non-HMR (non- MetS and non-NAFLD) and controls: 0.50 (0.08) vs. 0.48 (0.07) and 0.43 (0.06); *P* < 0.001	MetS components	8

ALT = alanine aminotransferase; AST = aspartate aminotransferase; BMI = body mass index; BMI-SDS = BMI- standard deviation score; BP = blood pressure; CCA = common carotid artery; CI = confidence interval; cIMT = carotid intima media thickness; CRP = C reactive protein; FMD = flow-mediated dilation; GGT = gamma-glutamyltranspeptidase; HDL = high-density lipoprotein cholesterol; HMR = high metabolic risk (MetS + NAFLD); HOMA-IR = homeostasis model of insulin resistance; IQR = interquartile range;IR = insulin resistance; LDL = low-density lipoprotein cholesterol; MetS = metabolic syndrome; MR = metabolic risk; NAFLD = nonalcoholic fatty liver disease; MRS = magnetic resonance spectroscopy; PWV = pulse wave velocity; SD = standard deviation; TC = total cholesterol; TG = triglycerides; WC = waist circumference.

**Table 2:** Study characteristics for nonalcoholic fatty liver disease and cardiac structure and function alterations as well as hypertension.

Authors	Study Design	Study Population (NAFLD vs. Non-NAFLD)	Diagnosis of NAFLD	Outcome Measures	Results	Adjustment Considered	NOS
Alp et al./ Turkey/2013 [33]	Cross-sectional	Hospital-based cohort of obese children and adolescents with (*n* = 93), and without (*n* = 307) NAFLD matched for gender and age; and control subjects recruited from subjects seen in hospital for minor illnesses (*n* = 150)	Liver ultrasound	LV structure and function (M-mode echocardiography; Pulsed and Tissue Doppler echocardiography)	Increased end-systolic thickness of the interventricular septum, LV mass and LV mass index as well as impaired LV systolic and diastolic functions were found in NAFLD group compared to non-NAFLD subjects and controls	Anthropometric variables, total fat mass, HOMA-IR	8
Singh et al./ USA/2013 [39]	Case-control	Hospital-based cohort of obese children and adolescents with (*n* = 15), and without (*n* = 15) NAFLD matched for age, Tanner stage, BMI *z* score, and percent body fat; and controls (*n* = 15) matched for age, and Tanner stage	MRS	LV structure and function (Integrated backscatter ultrasonography and speckle tracking echocardiography)	LV global longitudinal systolic strain and early diastolic strain rates were significantly decreased in obese children with NAFLD compared to both lean controls and obese subjects without NAFLD	Age, Tanner stage, BMI, percent body fat, intraabdominal adipose tissue volume, BP, TC, LDL, and insulin sensitivity indices	8
Sert et al./Turkey/ 2013 [32]	Cross-sectional	Hospital-based cohort of obese adolescents with (*n* = 97), and without (*n* = 83) NAFLD; and healthy lean subjects recruited from subjects seen in hospital for minor illnesses (*n* = 68)	Liver ultrasound and elevated ALT	LV structure and function (M-mode echocardiography; Pulsed and Tissue Doppler echocardiography)	Obese adolescents with NAFLD exhibited increased LV mass and LV mass index, as well as impaired LV diastolic function compared to both lean controls and obese subjects without NAFLD	Age, waist –to- hip ratio, BP, HOMA-IR, TC, HDL, AST and ALT levels	7
Pacifico et al./ Italy/2014 [40]	Cross-sectional	Hospital-based cohort of obese children and adolescents with (*n* = 54), and without (*n* = 54) NAFLD matched for age, gender, pubertal status, and BMI-SDS; and healthy control subjects (*n* = 18) matched for gender, age, and pubertal status	MRI; and liver biopsy in a subgroup of 41 NAFLD patients	LV structure and function (M-mode echocardiography; Pulsed and Tissue Doppler echocardiography)	Increased interventricular septum thickness at end-diastole and at end-systole, as well as impaired LV systolic and diastolic functions were found in NAFLD group. Children with more severe liver histology had worse cardiac dysfunction than those with milder liver changes	Age, gender, pubertal status, BMI-SDS, abdominal fat, BP, TG, HDL, WBISI	8
Schwimmer et al./2008/ [41]	Case-control	Hospital-based cohort of obese children and adolescents with (*n* = 150), and without (*n* = 150) NAFLD matched for age, gender, and BMI-SDS	Liver biopsy	Prevalence of elevated blood pressure	32% vs. 16%; *P* = 0.001		8
Pacifico et al./Italy/ 2010 [17]	Cross-sectional	Hospital-based cohort of obese children with (*n* = 100), and without (*n* = 150) NAFLD	Liver ultrasound and elevated ALT	Prevalence of elevated blood pressure	61% vs. 36.6%; *P* = 0.0001		8

ALT = alanine aminotransferase; AST = aspartate aminotransferase; BMI = body mass index; BMI-SDS = BMI-standard deviation score; BP = blood pressure; HDL = high-density lipoprotein cholesterol; HOMA-IR = homeostasis model of insulin resistance; LDL = low-density lipoprotein cholesterol; LV = left ventricular; MRI = magnetic resonance imaging; NAFLD = nonalcoholic fatty liver disease; MRS = magnetic resonance spectroscopy; TC = total cholesterol; TG = triglycerides; WBISI = whole body insulin sensitivity.

**Table 3:** Study characteristics for nonalcoholic fatty liver disease and type 2 diabetes.

Authors	Study Design	Study Population	Diagnosis	Outcome Measures Prediabetes/Diabetes	Results	NOS
Bedogni et al./ Italy/2012 [42]	Observational cohort	Hospital-based cohort of children and adolescents with BMI ≥ 95th[*n * = 571 with liver ultrasound assessment (234 with and 337 without NAFLD)]	Fasting glucose and/orOGTT	IFG, IGT, diabetes [Number (%) of cases]	Non-NAFLD vs. NAFLD: Normal/IFG/T2DM with fasting glucose, 337 (100)/0/0 vs. 229 (97.9)/5 (2.1)/0; *P* = 0.011 with OGTT, 311 (92.3)/24 (7.1)/2 (0.59) vs. 176 (75.2)/55 (23.5)/3 (1.3); *P* < 0.001	8
Boyraz et al./Turkey/2013 [43]	Observational cohort	Hospital-based cohort of pubertal obese children [*n * = 451 with liver ultrasound assessment (217 with and 234 without NAFLD)]	-	Diabetes [Number (%) of cases]	Non-NAFLD vs. NAFLD: 3 (1%) vs. 7 (3%); *P* < 0.01	8
Xanthakos et al./USA/2015 [44]	Observational cohort	Hospital-based cohort of adolescents undergoing bariatric surgery [*n * = 148 with intraoperative liver biopsy of whom 61 without NAFLD, 57 with NAFLD (not NASH), 21 with borderline NASH, and 9 with definite NASH]	-	Diabetes [Number (%) of cases]	Non-NAFLD vs. NAFLD (not NASH) vs. Borderline NASH vs. NASH: 4 (6.6%) vs. 5 (8.8%) vs. 5 (23.8%) vs. 6 (66.7%); *P* < 0.01	8
Pacifico et al./Italy/2015 [16]	Observational cohort	Hospital-based cohort of overweight/Obese children and adolescents [*n * = 158 with hepatic MRI assessment (80with and 78 without NAFLD)]	Fasting glucose and/orOGTT and/orHbA1c	Prediabetes, diabetes [Number (%) of cases]	Non-NAFLD vs. NAFLD: Prediabetes, 4 (5.1%) vs. 14 (17.5%); *P* < 0.025 Diabetes, 0 vs. 0	8

BMI = body mass index; IFG = impaired fasting glucose; IGT = impaired glucose tolerance; MRI = magnetic resonance imaging; NAFLD = nonalcoholic fatty liver disease; NASH = nonalcoholic steatohepatitis; OGTT = oral glucose tolerance test; T2DM = type 2 diabetes mellitus.

**Table 4:** Study characteristics for nonalcoholic fatty liver disease and low bone mineral density.

Authors	Study Design	Study Population (NAFLD vs. Non-NAFLD)	Diagnosis of NAFLD	Outcome Measures	Results	Adjustment Considered	NOS
Pirgon et al./Turkey/2011 [45]	Cross-sectional	Hospital-based cohort of obese children with (*n* = 42) and without (*n* = 40) NAFLD; lean controls seen in hospital for minor illnesses (*n* = 30)	Liver ultrasound and elevated ALT	LS BMD *z* score, mean (SD)	NAFLD vs. non-NAFLD and controls: 0.56 (0.3) vs. 1.02 (0.9) and 1.37 (1.04); *P* < 0.05	Age, gender, BMI-SDS, BP, lipids, HOMA-IR score	7
Pardee et al./USA/2012 [14]	Case-control	Hospital-based cohort of obese children with (*n* = 38) and without (*n* = 38) NAFLD matched for age, gender, race, ethnicity, and BMI	Liver biopsy	TB BMD *z* score, mean	NAFLD vs. non-NAFLD: −1.98 vs. 0.48; *P* < 0.0001	Age, gender, race/ethnicity, height, weight	8
Campos et al./Brazil/2012 [46]	Longitudinal	Hospital-based cohort of post-puberty obese adolescents with (*n* = 18) and without (*n* = 22) NAFLD	Liver ultrasound	TB BMD z score, mean (SD)	At baseline NAFLD vs. non-NAFLD: 1.5 ± 1.0 vs. 1.6 ± 1.0; *P* > 0.05. One-year of weight loss therapy resulted in an increase in total bone mineral content (but not in BMD) in youth with and without NAFLD	-	6
Pacifico et al./Italy/2013 [15]	Case-control	Hospital-based cohort of obese children with (*n* = 44) and without (*n* = 44) NAFLD matched for age, gender, pubertal stage and BMI-SDS	Hepatic MRI; and liver biopsy in a subset of 35 patients	LS and TB BMD z score, mean (95% CI)	NAFLD vs. non-NAFLD: LS, 0.55 (0.23–0.86) vs. 1.29 (0.95–1.63); *P* < 0.01 TB, 1.55 (1.23–1.87) vs. 1.95 (1.67–2.10); *P* = 0.06	Age, gender, pubertal stage, BMI-SDS	8
Chang et al./Korea/2015 [47]	Cross-sectional	Hospital-based cohort of 62 obese children with NAFLD (15 with simple steatosis and 47 with NASH); and 32 obese children with normal liver	Liver ultrasound and elevated ALT	TB age-matched BMD *z* score, median (range)	Simple steatosis and NASH vs. controls: 0.5 (1.2–1.9) and 0.6 (1.3–3.0) vs. 0.6 (1.3–1.5); *P* = 0.89	BMI, TC, percent trunk fat, 25(OH)D levels	6
Labayen et al./Spain/2018 [48]	Observational cohort	Hospital-based cohort of obese children with (*n* = 41) and without (*n* = 74) NAFLD	Hepatic MRI	TB BMD z score, mean (SD)	NAFLD vs. non-NAFLD: 0.94 (1.07) vs. 1.39 (0.94); *P* = 0.028	Gender, pubertal stage, total lean and fat masses, vitamin D/calcium intakes, physical activity	8

ALT = alanine aminotransferase; BMD = bone mineral density; BMI = body mass index; BMI-SDS = BMI-standard deviation score; BP = blood pressure; CI = confidence interval; HOMA-IR = homeostasis model of insulin resistance; LS = lumbar spine; MRI = magnetic resonance; NAFLD = nonalcoholic fatty liver disease; NASH = nonalcoholic steatohepatitis; SD = standard deviation; TB = total body; TC = total cholesterol.

**Table 5:** Study characteristics for nonalcoholic fatty liver disease and renal function alterations.

Authors	Study Design	Study Population (NAFLD vs. Non-NAFLD)	Diagnosis of NAFLD	Outcome Measures	Results	NOS
Manco et al./ Italy/2009 [49]	Case-control	Hospital-based cohort of children with NAFLD (*n* = 80) vs. controls admitted to hospital for minor surgery (*n* = 59) matched for age and gender	Liver biopsy	Albuminuria (mg/24 h), eGFR (creatinine clearance, mg/min)	No significant differences were found	7
Pacifico et al./ Italy/2016 [13]	Cross-sectional	Hospital-based cohort of obese children with (*n* = 268) and without NAFLD (*n* = 328); normal weight controls recruited from schools (*n* = 130)	Hepatic MRI	Microalbuminuria: 24-h albumin excretion rate, 30 mg to 299 mg Reduced eGFR: <90 mL/min/1.73 m^2^	NAFLD vs. non-NAFLD and controls: Number (%) of cases with microalbuminuria, 25 (9.3) vs. 13 (4.0) and 0; *P* < 0.0001 Number (%) of cases with reduced eGFR, 47 (17.5) vs. 22 (6.7) and 1 (0.77); *P* < 0.0001	8

eGFR = estimated glomerular filtration rate; MRI = magnetic resonance; NAFLD = nonalcoholic fatty liver disease.

**Table 6:** Study characteristics for nonalcoholic fatty liver disease and obstructive sleep apnea as well as well as polycystic ovary syndrome.

Authors	Study Design	Study Population (NAFLD vs. Non-NAFLD)	Diagnosis of NAFLD	Outcome Measures	Results	NOS
Verhulst et al./ Belgium/2009 [19]	Retrospective	Hospital-based cohort of overweight/obese children and adolescents (*n* = 75), of whom 25 with NAFLD	Elevated ALT and/or liver ultrasound	OSA defined by respiratory disturbance index (RDI) > 2.0 on polymnosonography	RDI was associated with NAFLD	7
Ayonrinde et al./ Australia/2016 [21]	Cross-sectional	Population cohort of adolescent girls (*n* = 199), of whom 16.1% with PCOS and 18.6% with NAFLD	Liver ultrasound	Prevalence of NAFLD in girls with PCOS	NAFLD was more prevalent in girls with PCOS than in those without (37.5% vs. 15.1%; *P* = 0.003)	8

NAFLD = nonalcoholic fatty liver disease; OSA = obstructive sleep apnea; PCOS = polycystic ovary syndrome.
